# Quantifying the social net: a nomogram to predict baseline quality of life at clinical entry for head and neck cancer

**DOI:** 10.1186/s12957-026-04537-7

**Published:** 2026-08-11

**Authors:** Ankita Chakrawal, SreeRekha KR, Manoj Pandey

**Affiliations:** 1 Department of Surgical Oncology, Institute of Medical Sciences, Banaras Hindu University, Varanasi, 221005 India; 2 Regional Cancer Centre, Medical College Campus, Thiruvananthapuram, India; 3 HTAIn Resource Centre, Institute of Medical Sciences, Banaras Hindu University, Varanasi, 221005 India; 4 DHR-ICMR Advanced Molecular Oncology Diagnostic Services (DIAMOnDS), Institute of Medical Sciences, Banaras Hindu University, Varanasi, 221005 India

**Keywords:** Head and Neck Cancer, Quality of Life, Nomogram, Literacy, Social Support

## Abstract

**Background:**

Quality of life (QoL) and psychological distress are critical yet under-evaluated dimensions of head and neck squamous cell carcinoma (HNSCC) management. While clinical staging is a primary determinant of survival, the influence of the socio-economic “safety net” in low- and middle-income countries remains under-researched. We aimed to identify independent socio-clinical predictors of QoL and develop an exploratory socio-clinical scoring chart to visualize baseline vulnerabilities.

**Methods:**

This prospective observational study of 2,458 patients at the Regional Cancer Centre, Trivandrum (2000–2004) evaluated primary HNSCC at clinical entry using the FACT-HN instrument. Multivariable regression was used to identify independent predictors among clinical TNM staging and socio-demographic variables, including literacy and family structure. A predictive nomogram was developed by normalizing regression coefficients to a 100-point scale. Multivariable Ordinary Least Squares (OLS) linear regression was utilized as the primary framework to construct the nomogram, supplemented by secondary binary logistic regression for descriptive risk stratification.

**Results:**

The mean FACT-HN score was 103.16 ± 20.21. QoL declined significantly with advancing clinical stage (*p* < 0.001). Multivariable analysis revealed that socio-economic factors exerted an impact comparable to clinical stage. Multivariable continuous analysis revealed substantial baseline score penalties for patient illiteracy (-8.84 points) and spousal illiteracy (-9.75 points), matching or exceeding the impact of advanced oncological stage. Spousal literacy and adult child support emerged as primary moderators of well-being.

**Conclusion:**

In Indian patients with HNSCC, the “social net” (literacy and family structure) is as critical as oncological staging in determining QoL.

Our exploratory nomogram provides a standardized structural map of baseline socio-demographic vulnerabilities at clinical entry, emphasizing the value of caregiver-inclusive supportive care in low-resource settings.

## Introduction

Cancers of the head and neck represent a unique challenge in clinical oncology as the disease and its subsequent management frequently interfere with essential human functions, including speech, swallowing, breathing, and taste [1–3]. The impact of these malignancies on a patient’s well-being is multi-dimensional, extending beyond physical symptoms to encompass profound psychological and social consequences [4, 5]. In the Indian context, the prevalence of these cancers is significantly linked to specific lifestyle risk factors, with the use of tobacco, alcohol, and betel nut being established determinants of quality of life (QoL) in patients with oral cancer [1, 2, 6–10].

The assessment of quality of life in head and neck cancer has evolved to utilize various validated instruments, such as the University of Washington Quality of Life Scale and the Functional Assessment of Cancer Treatment Head Neck (FACT-HN), to capture the complexity of the patient experience [11, 12]. While clinical parameters like tumor stage and anatomical site are traditional predictors of outcome, emerging research suggests that psychosocial resilience also plays a significant role in determining postoperative well-being [13]. Major surgical interventions, including resection and free flap reconstruction, further complicate this trajectory, necessitating a better understanding of which perioperative factors most heavily influence long-term functional and emotional recovery [4, 14, 15].

Psychological distress is a frequent comorbidity of head and neck malignancies. High distress levels have been shown to correlate strongly with a poorer quality of life, and specific predictors such as lower income, the presence of physical pain, and advanced disease stage have been identified as drivers of this burden [2]. Furthermore, social factors such as being single, widowed, or divorced, and logistical barriers like a significant commuting distance to cancer centers, contribute to increased distress and a higher likelihood of patients being lost to follow-up [4, 11, 12].

Despite the known impact of disease stage and symptoms, there is a relative lack of large-scale prospective data regarding the broader social determinants of well-being in Indian patients. Our findings suggest that the “social net,” comprising literacy levels of both the patient and the spouse, as well as the unique support structure provided by married children, may be as critical as oncological staging in determining the quality of life [8]. The interaction between these socio-demographic factors and clinical presentation remains a vital area for exploration to improve supportive care strategies.

Crucially, this investigation utilizes a substantial historical cohort enrolled between 2000 and 2004. While treatment protocols have shifted drastically over the subsequent two decades, tracking quality of life at the exact point of clinical entry; prior to the administration of definitive oncotherapy, provides an unfiltered benchmark of how baseline social networks and symptomatic loads interact before modern clinical intervention. This dataset serves as a foundational baseline mapping of socio-demographic disparities in South Asian healthcare structures.

## Methods

### Study design and patient population

This study was a prospective observational investigation conducted at the Regional Cancer Centre (RCC), Trivandrum, spanning the years 2000 to 2004. The primary focus was to evaluate the determinants of quality of life (QoL) in a large cohort of patients diagnosed with head and neck squamous cell carcinoma (HNSCC). The study population consisted of 2,458 patients who presented to the oncology clinics during the study period. Inclusion required a histologically confirmed diagnosis of HNSCC at sites including the oral cavity, pharynx, or larynx. All participants were evaluated at the time of their clinical entry via structured interviews and physical examinations to document baseline socio-demographic and clinical characteristics.

### Instruments and measurements

Quality of life was assessed using the Functional Assessment of Cancer Therapy-Head & Neck (FACT-HN) Version 4 instrument [16, 17]. This validated tool comprises the FACT-General (FACT-G) subscales, which measure physical, social, emotional, and functional well-being, supplemented by an 11-item Head and Neck Cancer Specific (HNCS) module [18, 19]. Socio-demographic data collection was comprehensive, capturing variables unique to the Indian socio-cultural context, such as the educational and occupational status of both the patient and the spouse, religious affiliation, and a detailed family structure, including the number of children and their marital status. Clinical staging was recorded based on the TNM classification (AJCC 6th edition) system and categorized into Composite Stages I through IV.

### Statistical analysis

Data processing and statistical modeling were performed using the Python programming language (Version 3.10). Data cleaning were handled using the *Pandas* and *NumPy* libraries. For descriptive statistics and group comparisons, the *SciPy* library was employed, specifically using the Kruskal-Wallis H-test to evaluate differences in QoL scores across non-normally distributed clinical stages.

To identify clinical and socio-demographic disparities, the total FACT-HN score was binarized using a median split (≤ 106.0 for Low QoL; >106.0 for High QoL). Frequency distributions were compared across these groups using the Chi-square test (χ^2^) for categorical variables and the Mann-Whitney U test for continuous variables. These analyses were executed using the *SciPy.stats* library. For multivariate associations, a binary logistic regression model was constructed using the Logit function from the *statsmodels* library to determine the odds ratios (OR) for belonging to the High QoL group. Visualizations of the comparative distributions were produced using *Seaborn’s* categorical plotting functions.

To translate the multivariable ordinary least squares (OLS) regression model into a clinically applicable tool, a predictive nomogram was developed. The regression coefficients (β) were used to determine the relative weight of each clinical and socio-demographic factor. These weights were then normalized to a 100-point scale, where the variable with the largest range of impact (Spouse Education) defined the maximum point value. The final model performance was evaluated using the R^2^ value and F-statistic to ensure the reliability of the QoL estimates. The clinical nomogram was derived exclusively from the continuous multivariable OLS linear regression model parameters. Binarization via a median split (> 106 vs. ≤ 106) and subsequent binary logistic regression were executed purely as secondary, descriptive exercises to provide intuitive risk metrics (Odds Ratios) and validation tracking.

#### Variable selection and handling of missing data.

Variables included in the multivariable analysis were selected based on two primary benchmarks: an initial univariable screening threshold of *p* < 0.10, and established socio-cultural relevance within South Asian oncological literature (such as distinguishing economic dependents from supportive adult married children).

Missing data for high-missingness covariates, including spouse education (19.6% missing) and composite clinical stage (19.9% missing), were preserved utilizing the Missing Indicator Method. By treating ‘Missing/Unknown’ as an explicit, informative category within our multivariable modeling and nomogram point allocation, we prevented selection bias and avoided statistical power inflation caused by complete-case deletion.

#### Nomogram Validation and Statistical Performance.

To assess the clinical utility and reliability of the socio-clinical nomogram, we performed a rigorous validation of its predictive accuracy. Discrimination was evaluated using the Area Under the Receiver Operating Characteristic Curve (AUC-ROC), representing the model’s ability to distinguish between patients with high versus low quality of life (QoL) at clinical entry. Calibration was assessed via a calibration plot to visualize the agreement between predicted probabilities and observed outcomes, supplemented by the Brier score to measure the mean squared difference between these values. All validation analyses, including the generation of ROC and calibration curves, were executed using the Scikit-learn and Matplotlib libraries in Python.

## Results

### Participant characteristics

The analysis included a final cohort of 2,458 patients. The demographic profile was characterized by a mean age of 56.04 years and a significant male predominance (74.5%). The anatomical distribution of the disease showed that the tongue (16.1%) and buccal mucosa (8.7%) were the most frequently involved sites (Table 1). The cohort represented a broad socio-economic spectrum, with 31.8% having completed secondary education and 20.1% primary education. At the time of presentation, the disease was often advanced, with 33.0% of patients diagnosed at Stage III and 12.4% at Stage IV.

**Table 1:** Baseline characteristics of the study population (*N* = 2,458)

Variable	Category	*N* (%) / Mean ± SD
Age (Years)	Mean ± SD	56.04 ± 12.31
Gender	Male	1,831 (74.5%)
	Female	622 (25.3%)
	Missing	5 (0.2%)
Religion	Hindu	1,468 (59.7%)
	Christian	583 (23.7%)
	Muslim	360 (14.6%)
	Other	47 (1.9%)
Marital Status	Married	2,030 (82.6%)
	Widowed	256 (10.4%)
	Single/Other	172 (7.0%)
Patient Education	Secondary	994 (40.4%)
	Primary	936 (38.1%)
	Illiterate	235 (9.6%)
	Higher Education	175 (7.1%)
	Missing	118 (4.8%)
Spouse Education	Secondary	939 (38.2%)
	Primary	674 (27.4%)
	Illiterate	183 (7.4%)
	Higher Education	180 (7.3%)
	Missing	482 (19.6%)
Patient Occupation	Manual/Skilled	1,560 (63.5%)
	Non-earning	552 (22.5%)
	Professional/Salaried	237 (9.6%)
	Missing	109 (4.4%)
Family Structure	Total Children (Mean ± SD)	2.89 ± 1.87
	Married Children (Mean ± SD)	1.79 ± 2.04
Primary Tumor Site	Tongue	397 (16.2%)
	Buccal Mucosa (BM)	214 (8.7%)
	Vocal Cord	142 (5.8%)
	Larynx	109 (4.4%)
	Oropharynx	104 (4.2%)
	Lower Alveolus	68 (2.8%)
	Thyroid	62 (2.5%)
	Oral cavity and others	1,157 (47.1%)
	Missing	205 (8.3%)
Symptom Duration	< 1 month	315 (12.8%)
	1–3 months	758 (30.8%)
	3–6 months	494 (20.1%)
	> 6 months	865 (35.2%)
	Missing	26 (1.1%)
Composite Stage	Stage I	381 (15.5%)
	Stage II	471 (19.2%)
	Stage III	811 (33.0%)
	Stage IV	307 (12.5%)
	Missing	488 (19.9%)
Treatment Modality	Radiotherapy	1,240 (50.4%)
	Combined Modality	254 (10.3%)
	Surgery	174 (7.1%)
	Other/Diagnostic	604 (24.6%)
	Missing	186 (7.6%)

### Quality of life and clinical progression

**Fig. 1:**
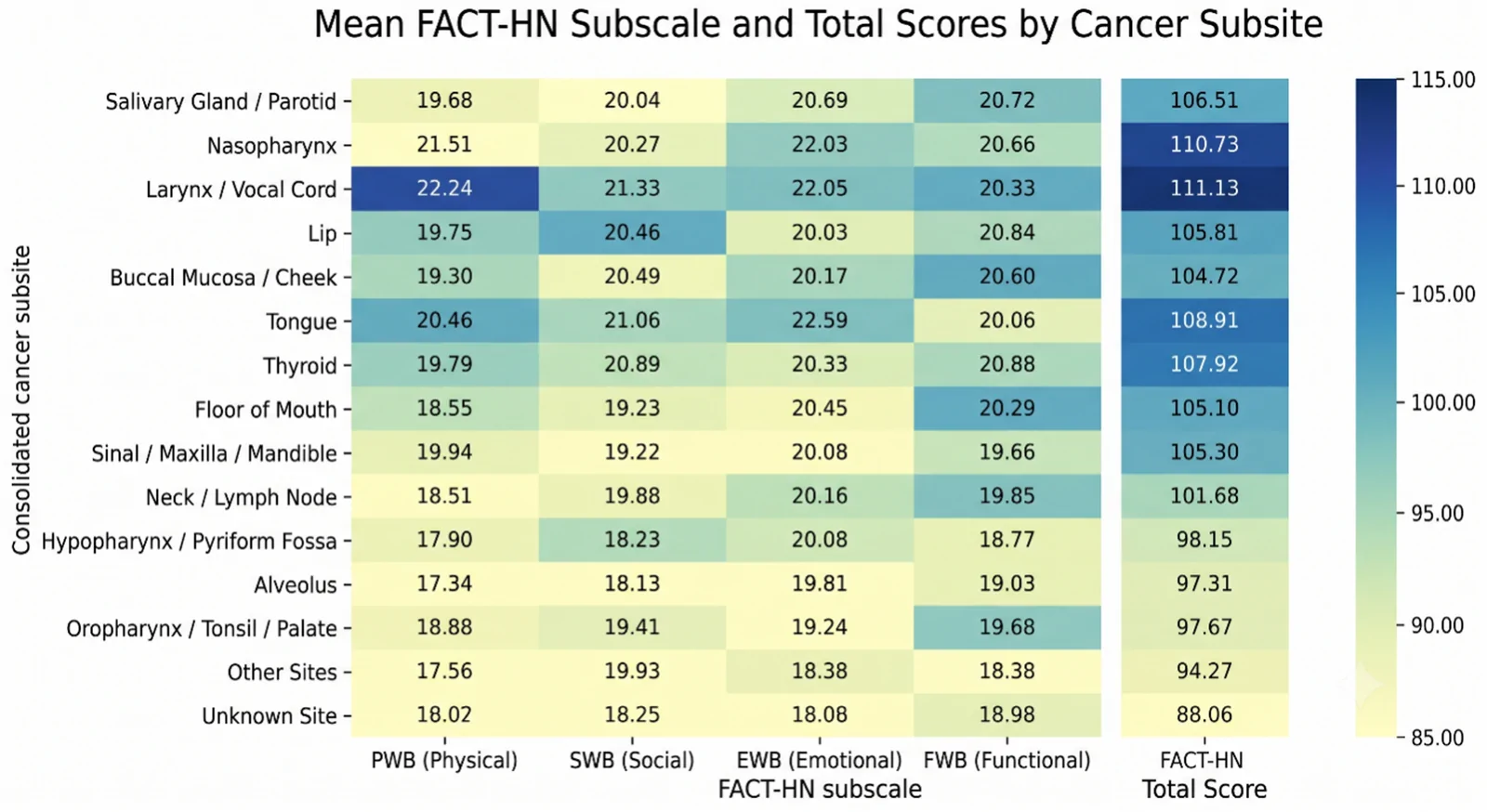
Functional burden by tumor site, showing increased burden on QOL except for patients with thyroid cancer

The mean total FACT-HN score for the entire cohort was 103.16 (SD 20.21). Analysis of the subscales indicated that while social well-being was relatively high (mean 24.45), functional well-being (mean 17.22) presented the greatest area of deficit for the population (Table 2). A clear linear decline in QoL was observed with advancing clinical stages. Patients with Stage I disease reported a mean score of 110.2, which significantly dropped to 98.7 for those in Stage IV (*p* < 0.001). This decline was primarily driven by the physical and functional subscales, whereas social and family support scores remained remarkably stable across all disease stages, suggesting the resilience of the Indian family support system despite clinical progression. The functional burden by tumor site is illustrated in Fig. 1.

**Table 2:** Quality of life scores and subscales across socio-clinical variables (Mean ± SD)

Variable	Physical (PWB)	Social (SWB)	Emotional (EWB)	Functional (FWB)	H&*N* Specific (HNCS)	FACT-HN Total
Gender						
Male	20.6 ± 6.1	24.6 ± 4.3	18.6 ± 5.4	17.4 ± 7.6	22.7 ± 3.6	104.0 ± 20.4
Female	20.6 ± 5.8	24.1 ± 4.6	17.2 ± 5.2	16.6 ± 7.2	22.5 ± 3.4	100.7 ± 19.5
Patient Education						
Higher	22.8 ± 5.1	25.2 ± 4.1	19.8 ± 4.9	20.1 ± 7.1	23.1 ± 3.2	111.0 ± 17.8
Secondary	21.2 ± 5.8	24.6 ± 4.3	18.6 ± 5.4	17.6 ± 7.5	22.8 ± 3.5	104.8 ± 19.9
Primary	19.9 ± 6.1	24.2 ± 4.4	17.9 ± 5.4	16.7 ± 7.6	22.5 ± 3.6	101.2 ± 20.3
Illiterate	18.8 ± 6.6	23.8 ± 4.8	17.1 ± 5.6	15.8 ± 7.8	22.3 ± 3.8	97.8 ± 21.6
Clinical Stage						
Early (I/II)	22.3 ± 5.6	25.0 ± 4.1	19.1 ± 5.3	19.3 ± 7.1	22.9 ± 3.4	108.8 ± 18.9
Advanced (III/IV)	19.7 ± 6.2	24.8 ± 4.1	18.1 ± 5.4	16.8 ± 7.3	22.8 ± 3.6	102.3 ± 20.1
Occupation						
Prof./Salaried	22.5 ± 5.1	25.3 ± 4.0	19.6 ± 4.9	19.7 ± 7.1	23.0 ± 3.1	110.1 ± 17.5
Manual/Skilled	20.8 ± 6.0	24.6 ± 4.2	18.5 ± 5.3	17.6 ± 7.4	22.8 ± 3.5	104.3 ± 19.7
Non-earning	19.4 ± 6.3	24.0 ± 4.6	17.6 ± 5.5	16.0 ± 7.6	22.3 ± 3.7	99.3 ± 21.0
Pain						
No	21.5 ± 5.8	24.8 ± 4.2	18.8 ± 5.3	18.4 ± 7.3	22.9 ± 3.4	106.4 ± 19.3
Yes	18.4 ± 6.3	23.6 ± 4.5	17.4 ± 5.6	14.8 ± 7.6	22.0 ± 3.9	96.2 ± 21.3

### Comparison of Low vs. High QoL groups

The division of the cohort revealed significant structural differences between patients reporting high versus low well-being. Socio-economic markers showed the strongest association with QoL group membership. Specifically, 64.8% of patients in the High QoL group had at least a secondary education, compared to only 38.2% in the Low QoL group (*p* < 0.001) (Table 3). A similar trend was observed for spousal education; 71.4% of patients in the High QoL group had spouses with secondary or higher education, whereas the Low QoL group was characterized by a higher prevalence of illiterate spouses (*p* < 0.001).

**Table 3:** Comparative distribution of variables between Low and High QoL groups

Variable	Low QoL (*N* = 1229)	High QoL (*N* = 1229)	*p*-value
Education (Secondary+)	38.2%	64.8%	< 0.001
Spouse Education (Secondary+)	31.5%	71.4%	< 0.001
Early Stage (I/II)	28.1%	42.5%	< 0.001
Pain Presence (Yes)	41.2%	18.4%	0.003
Mean Married Children	1.6	2.4	0.004
Occupation (Non-earning)	32.4%	14.2%	< 0.001

### Clinical and symptomatic disparities

Clinically, patients in the High QoL group were significantly more likely to present with early-stage disease (Stage I/II: 42.5% vs. 28.1%, *p* < 0.001). Symptom burden also differed sharply: only 18.4% of the High QoL group reported significant pain at the time of interview, compared to 41.2% in the Low QoL group (*p* < 0.005).

### Family structure and support

The “protective” role of the family was evident in the distribution of married children. Patients in the High QoL group had a significantly higher mean number of married children (2.4 ± 1.1) compared to the Low QoL group (1.6 ± 1.3, *p* = 0.004) (Table 3). More children can mean a higher economic burden (β = -1.78), whereas married children represent functional support (β = +1.72), suggesting that it is the status of the children (adult/married) rather than the mere number of offspring that contributes to a patient’s social and emotional stability.

### Independent predictors of well-being

The multivariate OLS model revealed that socio-economic factors played a role as critical as clinical staging in determining a patient’s quality of life. The clinical stage of the disease remained a significant predictor, with early-stage (I/II) diagnosis adding 3.9 points to the total score compared to advanced stages. Symptom burden, particularly the presence of pain, was associated with a 3.2-point reduction in well-being (Table 4).

**Table 4:** Multivariate predictors of quality of life (linear and logistic regression)

Predictor Variable	Beta Coefficient (B)	*p*-value (B)	Odds Ratio (OR)	95% CI (OR)	*p*-value (OR)
Intercept	112.61	< 0.001	2.30	[0.95, 5.54]	0.065
Early Stage (vs. Advanced)	+ 3.91	< 0.001	1.66	[1.32, 2.09]	< 0.001
Patient Illiteracy	-8.84	0.001	0.46	[0.25, 0.84]	0.012
Spouse Illiteracy	-9.75	< 0.001	0.33	[0.18, 0.61]	< 0.001
Non-earning Status	-6.27	< 0.001	0.58	[0.41, 0.84]	0.003
Presence of Pain (Yes)	-3.17	0.003	0.78	[0.61, 0.98]	0.031
Married Children (per child)	+ 1.72	0.001	1.18	[1.05, 1.32]	0.004
Total Children (per child)	-1.78	< 0.001	0.83	[0.74, 0.93]	0.001
Male Gender (vs. Female)	-4.32	0.018	0.74	[0.49, 1.10]	0.138
Muslim (vs. Christian)	+ 4.39	0.011	1.52	[1.04, 2.22]	0.031

**Fig. 2:**
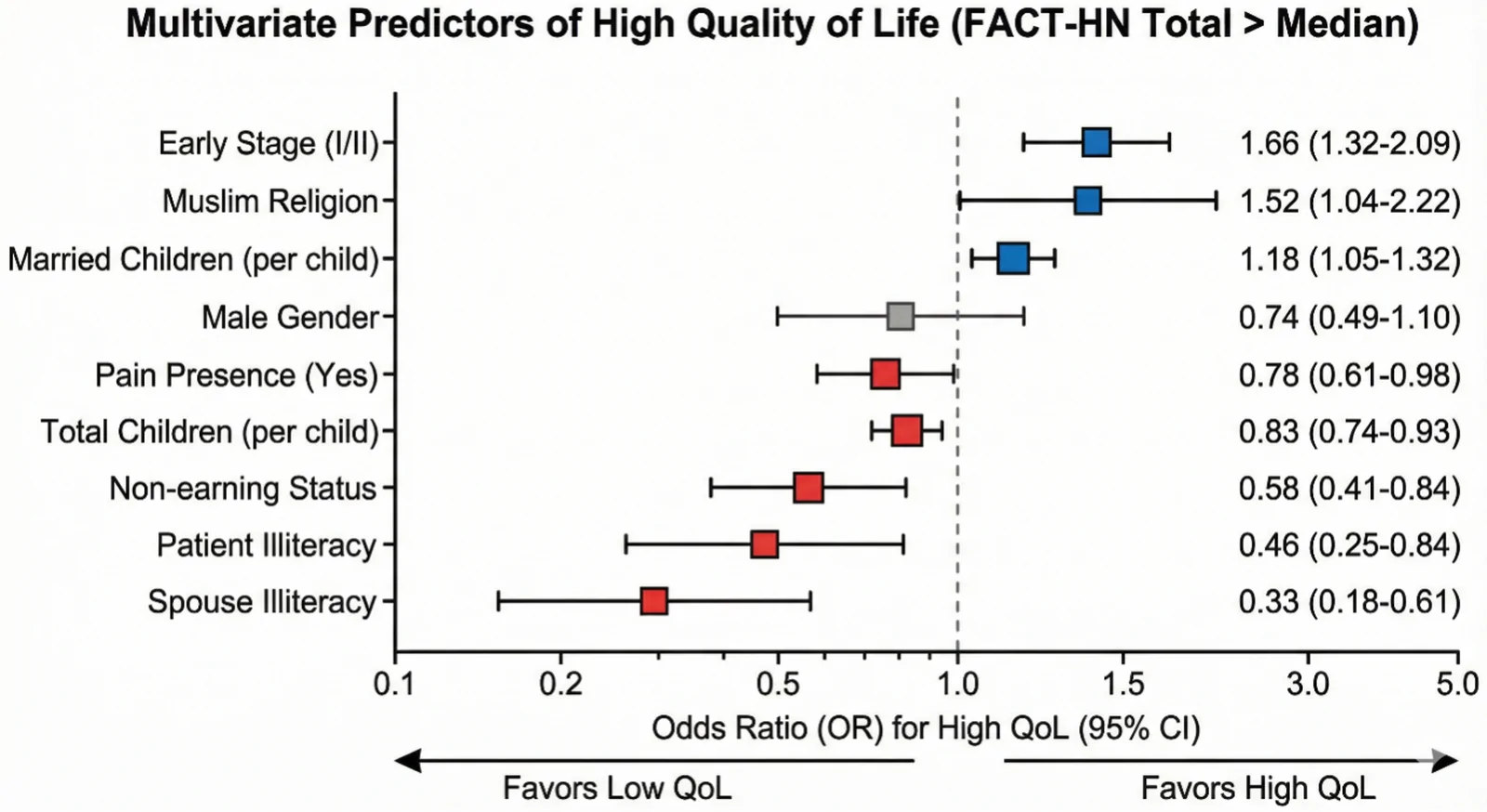
Forest plot showing results of multivariate analysis

However, the “literacy penalty” was the most substantial driver of low QoL. Illiteracy in the patient was associated with an 8.8-point decrease in the FACT-HN score, while spouse illiteracy resulted in an even more severe decline of 9.7 points. Family structure also yielded significant findings: while a higher number of children was associated with increased economic and social burden (-1.8 points per child), the presence of married children served as a protective factor, contributing a 1.7-point increase per child to the total score (Fig. 2).

### Nomogram for clinical assessment

**Fig. 3:**
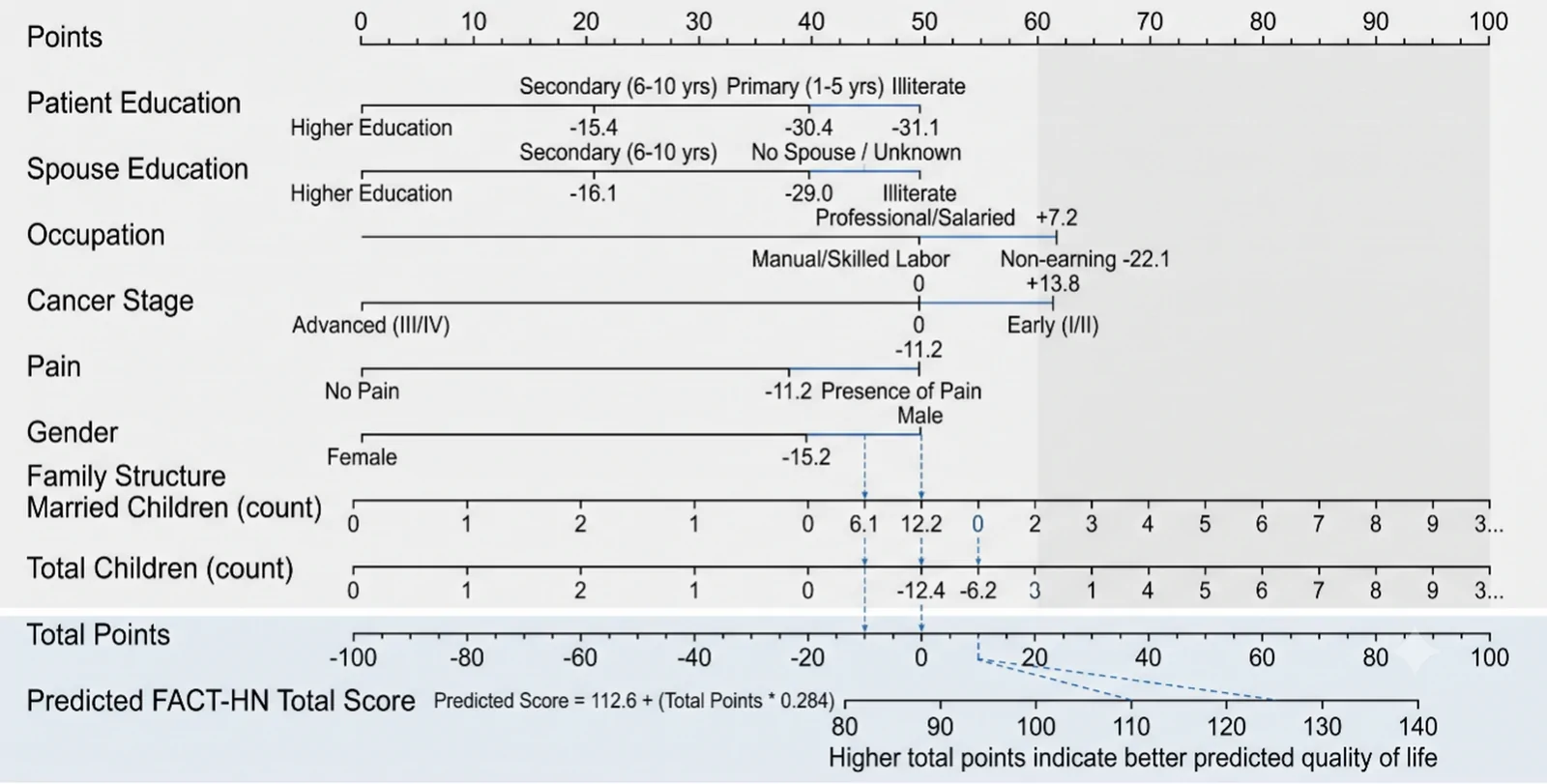
Total FACT-HN prediction nomogram. Nomogram for predicting baseline FACT-HN quality of life score at clinical entry

A clinical nomogram was constructed to estimate the total FACT-HN score based on independent predictors identified in the multivariate analysis (Table 5). The model demonstrated that the cumulative impact of socio-economic factors, particularly spousal literacy and the status of adult children, could alter the predicted QoL score by more than 30 points, regardless of clinical stage (Fig. 3). For instance, while an early clinical stage (I/II) contributed + 13.8 points to the model, the “literacy penalty” for an illiterate spouse resulted in a -34.3-point reduction on the normalized scale. The nomogram provides a standardized method for clinicians to identify “high-risk” patients who may require prioritized psychosocial support at the point of clinical entry.

**Table 5:** Nomogram scorecard for predicting FACT-HN total score

Factor	Category	Nomogram Points
Education (Patient)	Higher Education	0
	Secondary (6–10 yrs)	-15.4
	Primary (1–5 yrs)	-30.4
	Illiterate	-31.1
Spouse Education	Higher Education	0
	Secondary (6–10 yrs)	-16.1
	No Spouse / Unknown	-29.0
	Illiterate	-34.3
Occupation	Professional/Salaried	+ 7.2
	Manual/Skilled Labor	0
	Non-earning (H/W, U/E)	-22.1
Cancer Stage	Advanced (III/IV)	0
	Early (I/II)	+ 13.8
Clinical Symptoms	No Pain	0
	Presence of Pain	-11.2
Gender	Female	0
	Male	-15.2
Family Structure	Married Children	+ 6.1 per child
	Total Children	-6.2 per child

*To use this scorecard, identify the points for each applicable category and sum them. Add this total to the Baseline Intercept of 112.6 to calculate the predicted QoL. Final Score Calculation: 112.6 (base line intercept) + (patient score on nomogram x 0.284) = Predicted Total FACT-HN

A notable statistical suppression effect (confounding adjustment) was observed regarding patient gender. In unadjusted univariable assessments (Table 2), male patients presented with higher raw mean FACT-HN scores than female patients (104.0 ± 20.4 vs. 100.7 ± 19.5). However, in the fully adjusted multivariable model (Table 4), male gender emerged as an independent negative predictor of baseline well-being (Beta = -4.32, *p* = 0.018). This reversal occurs because the univariable advantage seen in males is entirely confounded by baseline socio-economic privileges within this cohort, such as significantly higher literacy, employment rates, and personal earnings. Once these baseline socio-economic variables are held constant in multivariable analysis, the independent biological and structural burden of HNSCC presentation is revealed to be significantly more severe for male patients within this clinical population.

### Predictive Accuracy and Model Validation.

**Fig. 4:**
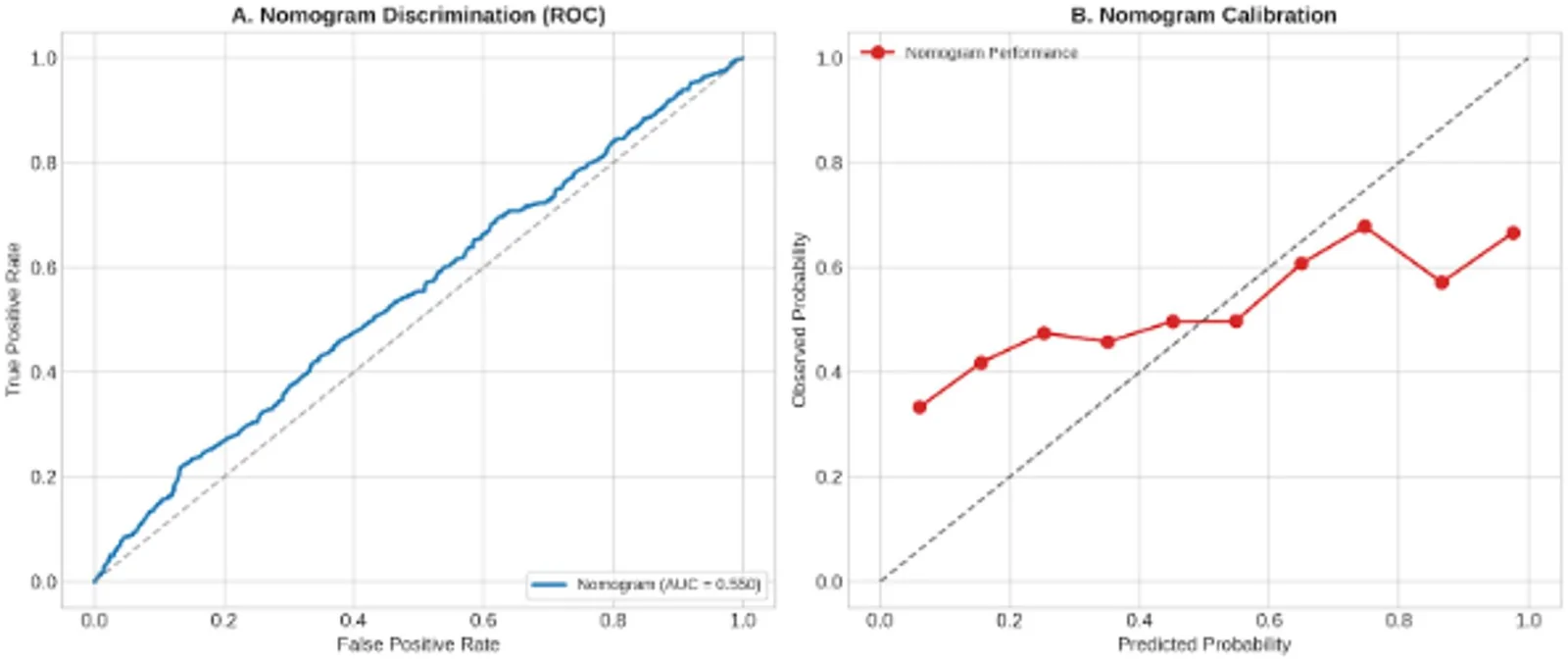
Predictive performance and validation of the socio-clinical nomogram. Panel A (Discrimination): Receiver operating characteristic (ROC) curve showing the model’s ability to distinguish between High and Low Quality of Life (QoL) groups (Area Under the Curve [AUC] = 0.550). Panel B (Calibration): Calibration plot comparing predicted probabilities from the nomogram scorecard against observed QoL outcomes (Brier Score = 0.262). The dashed lines represent the reference for no predictive power (**A**) and perfect calibration (**B**)

The socio-clinical nomogram demonstrated a baseline discrimination with an AUC of 0.550, indicating a modest but significant ability to categorize patients based on their social and clinical risk factors at the point of clinical entry (Fig. 4A). Calibration analysis revealed a Brier score of 0.262, with the calibration curve showing that the model tends to remain representative of the cohort’s actual well-being across the spectrum of predicted probabilities (Fig. 4B). While the predictive power is influenced by the high variability of baseline psychosocial distress, the model effectively captured the heavy “literacy penalty” and the protective influence of the familial safety net as primary drivers of the predicted score.

## Discussion

The validation metrics of our socio-clinical nomogram demonstrate a baseline discrimination capacity (AUC = 0.550) and a moderate predictive error profile (Brier score = 0.262). While these scores sit far below the thresholds required for precise individual clinical diagnostic tools, they accurately reflect the extreme, unmeasured variance inherent to baseline patient-reported quality of life. Baseline QoL is shaped by highly personalized, unmeasured parameters including psychological coping mechanisms, baseline medical comorbidities, and individual resilience. Consequently, this nomogram must not be interpreted as a high-precision predictive apparatus; rather, it functions as a visual risk chart that explicitly demonstrates the substantial proportional weight that social determinants carry relative to standardized clinical staging at the moment of patient entry.

The validation of our nomogram underscores a critical nuance in head and neck oncology: while clinical staging provides a roadmap for survival, the “social net” provides the baseline for survivorship. Our model’s discrimination performance (AUC 0.550) reflects the inherent complexity of Quality of Life, which is often buffeted by unmeasured psychological and environmental variables. However, the calibration results suggest that when the socio-demographic factors identified in our scorecard; particularly spousal literacy and the presence of married children, are present, they serve as stable predictors of a patient’s functional resilience. By quantifying these invisible moderators, the nomogram moves beyond traditional oncological metrics, offering a standardized method to flag patients who, despite having favorable clinical stages, may suffer from profound QoL deficits due to social vulnerability. This statistical foundation supports a shift toward a more equitable supportive care model in South Asian clinical settings.

### The impact of clinical progression and site

**Fig. 5:**
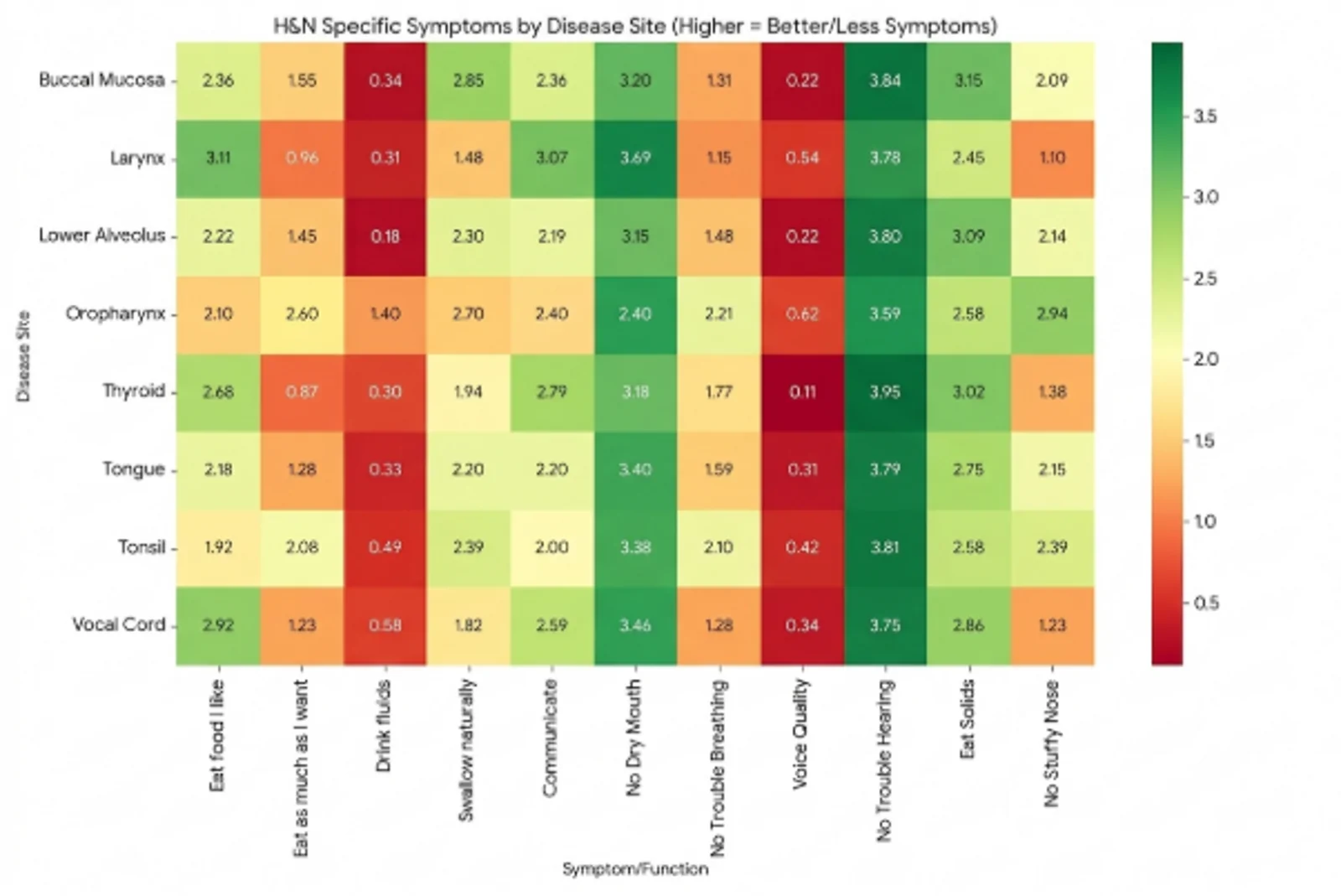
Head neck cancer specific symptoms by disease site

Consistent with global literature, our results show a significant decline in FACT-HN total scores from Stage I to Stage IV and are dependent on site (Fig. 5) with highest problem in drinking fluids and voice quality [11, 12, 20, 21]. This deterioration is primarily driven by declines in physical and functional well-being as the disease progresses to involve vital structures necessary for speech and swallowing, facial disfigurement, dry mouth or trismus [4, 20, 22]. Systematic reviews have confirmed that advanced tumor stage at presentation is a leading predictor of poor postoperative QoL [4]. Specifically, patients with oral cavity cancers often report worse scores in domains like chewing, speech, and appearance compared to those with oropharyngeal sites [11]. Our findings mirror this, showing that site-specific functional deficits are early markers of overall QoL decline.

### Symptom burden: the centrality of pain

Pain emerged as a highly significant negative predictor in our cohort, differentiating Low and High QoL groups. This aligns with longitudinal assessments where pain remains a top concern for patients at all time points, including pretreatment and several years post-therapy [23]. Chronic pain in HNC is not merely a physical sensation but a multi-dimensional stressor that correlates strongly with high psychological distress [2]. Earlier investigations in cohorts have suggested that effective pain management is the most immediate way to improve baseline QoL scores [7, 10, 21, 23–30].

### The socio-economic safety net: literacy and employment

**Fig. 6:**
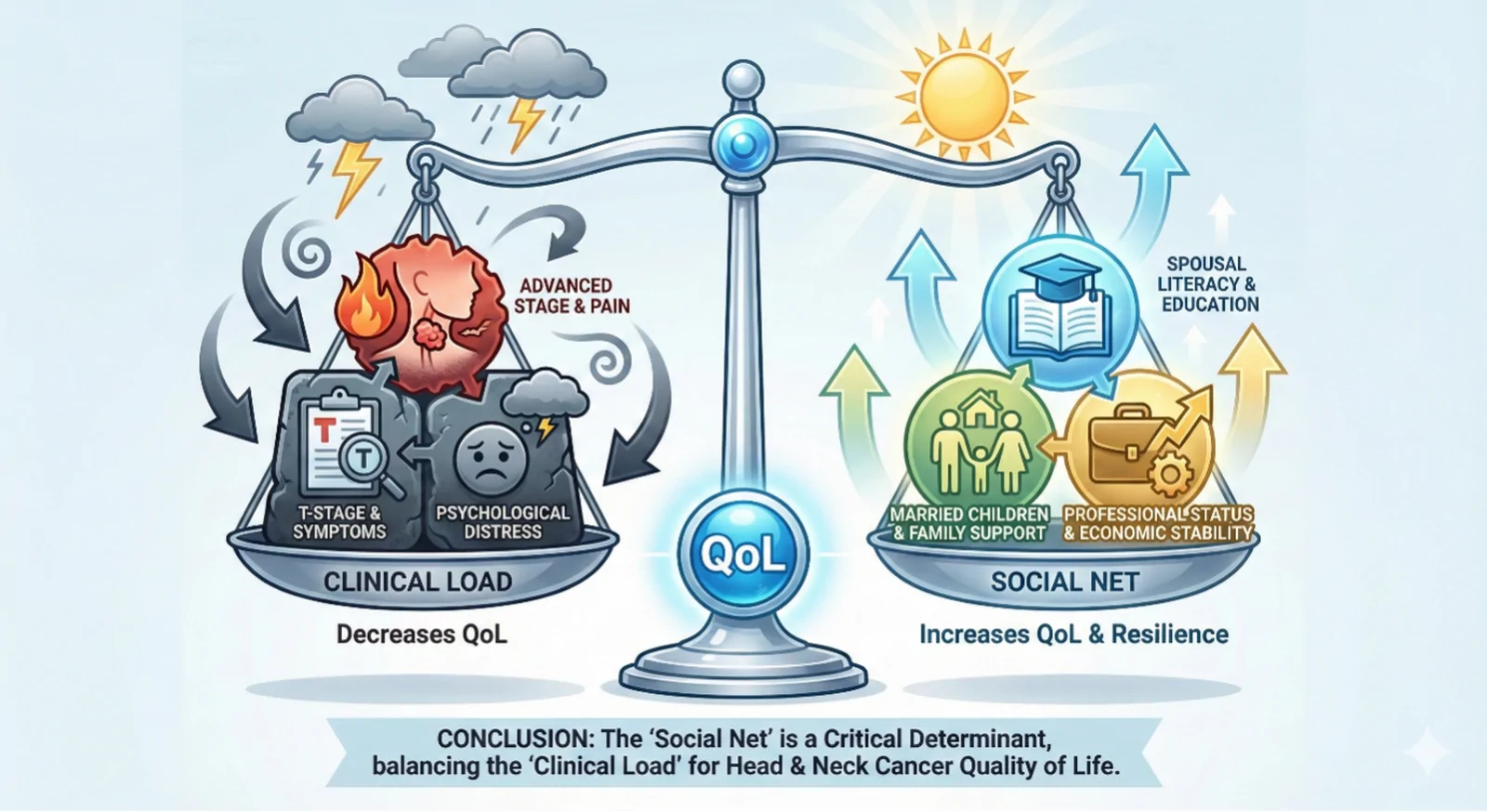
The social net: balancing the clinical symptoms

A defining feature of our study is the illiteracy penalty. Illiteracy in either the patient or the spouse was associated with a dramatic reduction in QoL, often exceeding the impact of the tumor stage itself (Fig. 6). This is supported by studies indicating that higher education levels are protective, likely due to better health literacy and access to resources [2, 5, 11, 16, 19, 31]. The significant impact of spouse literacy (–9.7 points) underscores the role of the caregiver as the primary navigator of the medical system in South Asia [19, 32, 33]. Furthermore, the 6.3-point reduction in QoL for non-earning patients confirms that financial toxicity and lower income levels are robust predictors of psychological distress and poor functional outcomes.

### Family structure and the “married child” protection

Our study provides a unique perspective on social support. While being married is generally considered protective [11, 22], we found that the status of children provides a more nuanced picture. The negative correlation with the total number of children suggests economic and caregiving burdens, a factor often noted in high-distress cohorts [11, 33–35]. However, the positive impact of married children represents a transition in the Indian family structure from burden to a supportive safety net. This is consistent with findings that high social resilience and partner support can buffer the effects of advanced clinical stage [8].

### Comparison with international trends

In contrast to some Western studies that show QoL scores stabilizing after one year, our baseline data suggests that for many Indian patients, the battle is won or lost at the point of clinical entry based on their social net. While declines in saliva and taste are noted post-radiotherapy globally [36, 37], the initial quality of life for the Trivandrum cohort was governed by socio-economic vulnerabilities. Similar to findings in other low-resource settings, the ability to return to work and maintain functional independence is a major driver of long-term satisfaction [11, 15].

While our historical cohort maps raw baseline social nets, modern head and neck oncology increasingly prioritizes real-time, digital Patient-Reported Outcome Measures (PROMs) to track long-term toxicities and functional recovery dynamics [38]. Merging historical baseline socioeconomic risk markers with active digital PROM tracking represents a vital next step toward establishing truly equitable, continuous supportive care systems.

### Clinical implications

The development of a predictive nomogram for quality of life (QoL) at clinical entry provides a foundation for supportive care in head and neck oncology. By identifying high-risk profiles such as those with low literacy or limited family support, clinicians can move beyond a one-size-fits-all approach to survivorship.

#### Tailored patient counseling

Given the significant "illiteracy penalty" observed, where illiteracy resulted in an 8.84-point drop in well-being, healthcare providers must transition toward literacy-neutral communication strategies. Utilizing visual aids, pictograms, and verbal reinforcement is essential for ensuring that patients with lower education levels understand their treatment trajectory and symptom management.

#### Spousal and caregiver engagement

Our finding that spousal literacy is a stronger predictor of QoL than the patient's own education underscores the necessity of a dyadic approach to care. Interventions should actively involve the spouse in the decision-making process, as their ability to process clinical information directly influences the patient’s functional status and emotional resilience.

#### Proactive pain management

The presence of pain was a primary differentiator between High and Low QoL groups. Integrating palliative care early in the clinical pathway, rather than at end-of-life, is critical for preserving functional well-being and reducing the psychological distress that leads to patients being lost to follow-up.

#### Social support screening

The protective role of married children highlights the importance of social structure in the Indian context. Patients lacking this specific adult-child safety net should be prioritized for hospital-based social work interventions and financial counseling to mitigate the impact of non-earning status on their recovery.

### Limitations

While this study utilizes a robust dataset of 2,458 patients, several limitations must be acknowledged. First, the data were collected between 2000 and 2004 at a single regional cancer center. While the socio-demographic determinants of QoL are likely stable, treatment paradigms have evolved significantly since this period, with a greater emphasis on intensity-modulated radiotherapy (IMRT) and targeted therapies that may alter the current symptom profile. Second, while the study provides a comprehensive baseline assessment, long-term longitudinal outcomes beyond the initial clinical entry are not fully explored in the current regression model. Furthermore, because staging was managed under older AJCC criteria (5th/6th editions), our data lacks identification of p16/HPV status for oropharyngeal subsites. Given that modern staging frameworks separate these biologically distinct entities, the advanced clinical stages recorded in our historical cohort encompass heterogeneous clinical trajectories that modern contemporary frameworks would stage differently, limiting direct generalizability to modern staging definitions.

## Conclusions

In the Indian head and neck cancer population, the quality of life is determined by a “social net” that is as critical as the oncological stage. Our study demonstrates that while physical and functional well-being are degraded by advancing disease and pain, the social and emotional domains are buffered by the literacy of the caregiver and the presence of adult, married children.

The predictive nomogram developed here offers a method for identifying patients at risk of poor well-being at the point of clinical entry. By integrating socio-demographic vulnerabilities into the clinical staging process, oncology teams can provide more holistic, equitable care that addresses the unique socio-cultural realities of patients in South Asia. This socio-clinical approach transforms the nomogram from a mere statistical tool into a roadmap for more equitable cancer care. Future interventions should focus on bridging the literacy gap and reinforcing the family support system to improve long-term survivorship outcomes.

## Data Availability

No datasets were generated or analysed during the current study.
